# Pharmacogenetic association of diabetes-associated genetic risk score with rapid progression of coronary artery calcification following treatment with HMG-CoA-reductase inhibitors —results of the Heinz Nixdorf Recall Study

**DOI:** 10.1007/s00210-021-02100-7

**Published:** 2021-05-22

**Authors:** Sonali Pechlivanis, Dominik Jung, Susanne Moebus, Nils Lehmann, Amir A. Mahabadi, Per Hoffmann, Raimund Erbel, Markus M. Nöthen, Hagen S. Bachmann

**Affiliations:** 1 Institute of Pharmacology and Toxicology, Centre for Biomedical Education and Research, Witten/Herdecke University, Witten, Germany; 2 Institute of Epidemiology, Helmholtz Zentrum München - German Research Center for Environmental Health, Neuherberg, Germany; 3 Centre for Urbane Epidemiology, University Hospital Essen, Essen, Germany; 4 Institute for Medical Informatics, Biometry and Epidemiology, University Hospital Essen, Essen, Germany; 5 Department of Cardiology and Vascular Medicine, West German Heart and Vascular Center, University Hospital Essen, Essen, Germany; 6 Division of Medical Genetics, Department of Biomedicine, University of Basel, Basel, Switzerland; 7 Institute of Human Genetics, School of Medicine & University Hospital Bonn, University of Bonn, Bonn, Germany

**Keywords:** HMG-CoA-reductase inhibitors, Pleiotropic effects, Diabetes mellitus, Genetic risk score, Progression of coronary artery calcification, Cohort study

## Abstract

HMG-CoA-Reductase inhibitors (HMGRIs) are currently the most widely used group of drugs in patients with coronary artery disease (CAD) and are given preemptively to patients with high levels of cholesterol, including those with diabetes mellitus (DM). However, intake of HMGRIs also increases the progression of coronary artery calcification (CAC) and the risk of developing DM. This study aimed to investigate whether HMGRI intake interacts with the diabetes-associated genetic risk score (GRS) to affect CAC progression using data from the population-based Heinz Nixdorf Recall (HNR) study. CAC was measured in 3157 participants using electron-beam computed tomography twice, at baseline (CAC_b_) and 5 years later (CAC_5y_). CAC progression was classified as slow, expected, or rapid based on predicted values. Weighted DM GRS was constructed using 100 diabetes mellitus–associated single nucleotide polymorphisms (SNPs). We used log-linear regression to evaluate the interaction of HMGRI intake with diabetes-associated GRS and individual SNPs on CAC progression (rapid vs. expected/slow), adjusting for age, sex, and log(CAC_b_ + 1). The prevalence of rapid CAC progression in the HNR study was 19.6%. We did not observe any association of the weighted diabetes mellitus GRS with the rapid progression of CAC (relative risk (RR) [95% confidence interval (95% CI)]: 1.01 [0.94; 1.10]). Furthermore, no indication of an interaction between GRS and HMGRI intake was observed (1.08 [0.83; 1.41]). Our analyses showed no indication that the impact of HMGRIs on CAC progression is significantly more severe in patients with a high genetic risk of developing DM than in those with a low GRS.

## Introduction

Atherosclerosis is the primary cause of coronary artery disease (CAD) and precedes the onset of coronary heart disease (CHD) by decades (Erbel & Budoff 2012; McClelland et al. 2015; Möhlenkamp et al. 2011). Coronary artery calcification (CAC) is one of the most sensitive and specific markers of coronary atherosclerosis, and the quantification of CAC has been shown to improve the ability to predict future CHD events (Budoff et al. 2010; Elias-Smale et al. 2010; Erbel & Budoff 2012; Lehmann et al. 2018; McClelland et al. 2015; Möhlenkamp et al. 2011; Taylor et al. 2008).

HMG-CoA-Reductase inhibitors (HMGRIs; also commonly referred to as *statins*) are the most widely used lipid-lowering medication for the majority of people with CHD risk, including those with diabetes mellitus type 2 (DM) (Knuuti et al. 2020). They are strongly recommended in many primary prevention guidelines from the European Association for Cardiovascular Prevention and Rehabilitation (EACPR) and the American College of Cardiology/American Heart Association (ACC/AHA) ("JBS 2: Joint British Societies' guidelines on prevention of cardiovascular disease in clinical practice," 2005; Piepoli et al. 2016; Robson 2008). However, recent results from an observational study and a clinical trial indicate that HMGRIs promote the progression of CAC (Dykun et al. 2016; Henein et al. 2015). Likewise, earlier studies concluded that HMGRI intake does not halt progression of CAC and enhances it in type 2 diabetes patients (Anand et al. 2007; Houslay et al. 2006; Saremi et al. 2012; Terry et al. 2007). Other studies have shown that HMGRIs promote coronary atheroma calcification and are implicated in the calcification of vascular smooth muscle cells as well as mesenchymal cells (Kupcsik et al. 2009; Puri et al. 2015; Trionet al.  2008).

**Fig. 1:**
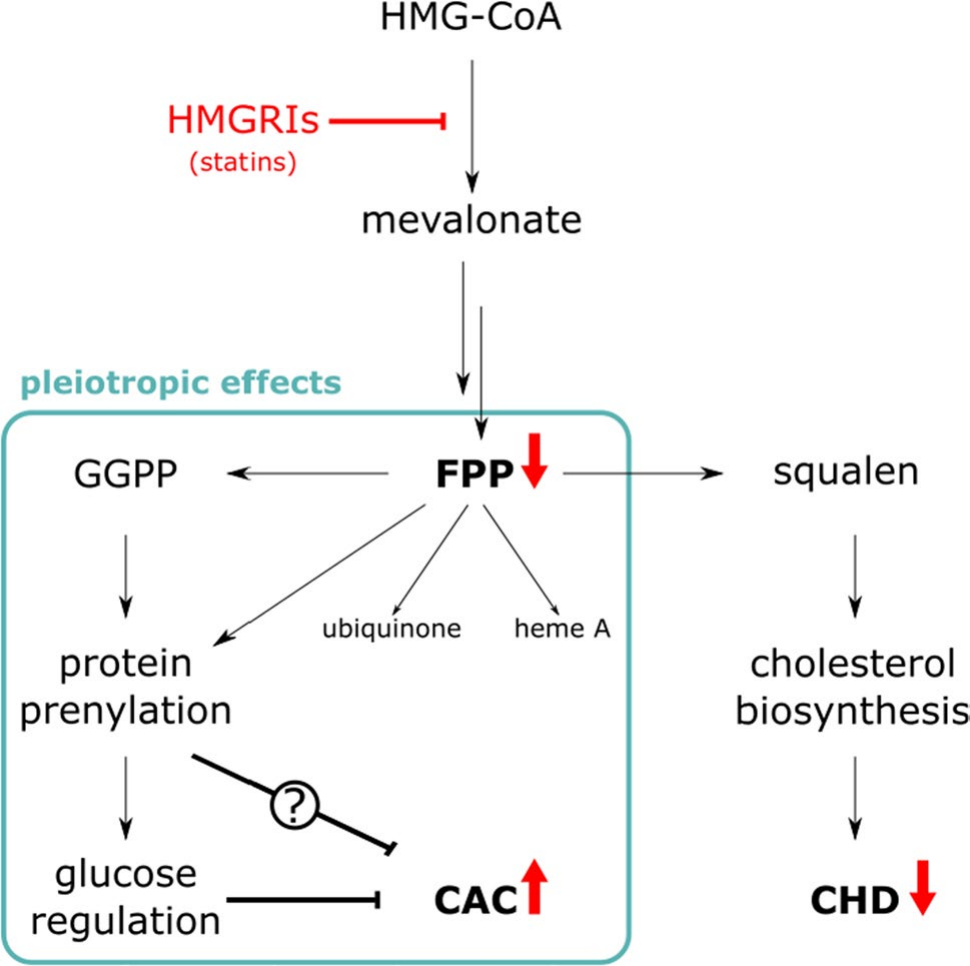
HMG-CoA-Reductase inhibitors (HMGRIs; formerly known as statins) lead to reduced FPP levels, which is the reason for reduced cholesterol synthesis and the rationale for their use in the prevention of coronary heart disease (CHD). Additionally, HMGRIs exhibit pleiotropic (i.e., cholesterol synthesis independent) effects, some of which may counteract their desired LDL-cholesterol lowering effect. Red arrows indicate up- and downregulation upon HMGRI treatment

HMGRIs bind directly to 3-hydroxy-3-methylglutaryl CoA (HMG-CoA) reductase within the mevalonate pathway to impair endogenous cholesterol synthesis, thus lowering LDL-cholesterol levels (Fig. 1). Problems may arise from the “pleiotropic effects” of HMGRIs, a collective term describing the multitude of (sometimes unintentional and often less well understood) HMGRI effects independent of cholesterol synthesis. They are mostly mediated through altered levels of the mevalonate pathway’s isoprenoid intermediates farnesyl pyrophosphate (FPP) and geranylgeranyl pyrophosphate (GGPP) (Fig. 1). For instance, heme A and ubiquinone, both of which are involved in oxidative phosphorylation, are derived from isoprenoid precursors. The available data suggest that HMGRIs modulate redox systems that are implicated in the development of atherosclerosis (Okuyama et al. 2015; Rhee et al. 2015).

Other pleiotropic effects originate from reduced protein prenylation, a posttranslational modification involving the direct transfer of FPP or GGPP, which is crucial for the proper function of hundreds of proteins. There is evidence that prenylated proteins play an important role in the pathogenesis of diabetes and the regulation of glucose levels (Kowluru & Kowluru 2015). At the same time, several observational studies have suggested an association between HMGRIs and elevated risks of developing DM (Carter et al. 2013; Cederberg et al. 2015; Waters et al. 2011). Furthermore, several studies indicated an association of DM and, more specifically, poor glycemic control with CAC progression (Koulaouzidis et al. 2013; Snell-Bergeon et al. 2003).

Recent large-scale genome-wide association studies (GWASs) and meta-analyses have identified 100 genetic variants that are associated with DM (Locke et al. 2015; Mahajan et al. 2014; Scott et al. 2017; Zhao et al. 2017). Moreover, it is not known whether genetic variation at most of these loci exacerbates the effects of HMGRIs on the risk of progression of CAC. A desirable clinical goal is to incorporate genetic information in the form of a genetic risk score (GRS) for DM. This would improve the predictive power of a model when compared with a model consisting of only known (lifestyle) risk factors in asymptomatic individuals so that preventive measures, if available, can be taken.

It is important to preclude a disproportionately high risk of developing DM or intense CAC in particular patient groups treated with HMGRIs. The necessity of such investigations is highlighted by three facts: (i) the widespread use of HMGRIs, (ii) the fact that individuals under HMGRI therapy have been shown to have higher CAC as well as a higher risk of developing DM, and (iii) the evidence that DM is associated with CAC progression. To this end, we investigated the putative interaction between the DM GRS (constructed using GWAS-identified genetic variants) and HMGRI intake concerning its influence on the progression of coronary artery calcification.

## Materials and methods

### Study population

At baseline (b), 4814 participants aged between 45 and 75 years (50% women) from the Heinz Nixdorf Recall Study (Risk Factors, Evaluation of Coronary Calcium, and Lifestyle) were randomly selected from the registration lists of the densely populated Ruhr metropolitan cities in Germany (residents of Essen, Bochum, and Mülheim) between December 2000 and August 2003. The rationale and design of the study were previously described in detail (Schmermund et al. 2002). The Heinz Nixdorf Recall Study is an already well-described population-based cohort study (Stang et al. 2005). Data of the study participants have been repeatedly used to address research questions of different medical fields (Heilmann-Heimbach et al. 2017; Locke et al. 2015; Malhotra et al. 2019; Orban et al. 2017; Shungin et al. 2015; Stang et al. 2007; Thanassoulis et al. 2013; Tzivian et al. 2016). To avoid identification and profiling of the participants, strict data protection is applied. However, for the purpose of replication, other researchers are allowed to access data upon request, which is the same way the authors of the present paper obtained the data. Data requests can be addressed to recall@uk-essen.de. The first follow-up examination took place 5 years after the baseline examination, and the participants were re-invited to attend.

The study participants were selected based on several exclusion and inclusion criteria. First, we excluded participants with prior CAD (coronary artery bypass surgery and/or interventional revascularization and a history of prior myocardial infarction) (n = 327) at baseline. Of the remaining participants, only the participants with CAC measurements at baseline (CAC_b_) and first follow-up (CAC_5y_) were included (approximately 5 years apart, 5.1 ± 0.3 years) (n = 3675). Additionally, the following groups of participants were excluded: (i) participants with stent implementation, bypass, balloon dilatation, or myocardial infarction during the 5-year follow-up (n = 154); (ii) participants outside the study age range (45–74 at baseline, 50–79 at the 5-year follow-up, n = 12); and (iii) participants with missing Framingham risk factors information (n = 28) (Erbel et al. 2014; Lehmann et al. 2016, 2018).

Lymphocyte DNA was isolated from EDTA anticoagulated venous blood using a Chemagic Magnetic Separation Module I (Chemagen, Baesweiler, Germany). Genotyping was performed using different Illumina microarrays (Metabochip, Omni1-Quad, OmniExpressv1.0, HumanCoreExome (v1.0 and v1.1); Illumina, San Diego, USA) according to the manufacturer’s protocols. Quality control was applied prior to the imputation, separately for each chip, and was first performed on the subject level including sex, ethnicity, and relatedness checks, excluding subjects with missing genotype data > 5%. Furthermore, single nucleotide polymorphisms (SNPs) with a minor allele frequency (MAF) < 1%, a missing genotype frequency > 5%, or a deviation from Hardy–Weinberg Equilibrium (HWE) (*p* < 10^−5^) were excluded. Imputation was carried out using IMPUTE v.2.3.1 with reference data from 1000 Genomes Phase 1, release March 2012, for the Metabochip and 1000 Genomes Phase 3, release October 2014, for all other microarray data (Frank et al. 2019; Geisel et al. 2016; Pechlivanis et al. 2020, 2013). The imputed data were then converted to the PLINK ped format using the threshold ≥ 0.8 in GTOOL v0.7.5.

For the present study, data from 3157 participants was used. The participants were included with CAC measurements at two points of time (CAC_b,_ CAC_5y_), and no missing information on age, sex, genetic risk score, and intake of HMGRIs. The study was approved by the ethical committees at the University Hospital of Essen, Germany, and was conducted in accordance with the principles expressed in the Declaration of Helsinki. The study was certified and recertified according to DIN EN ISO 9001:2000/2008. All study participants gave their written informed consent.

### Genetic risk scores

The SNPs for the GRS were selected from the published diabetes mellitus GWASs (Gaulton et al. 2015; Mahajan et al. 2014; Scott et al. 2017; Zhao et al. 2017). The average weighted GRS for each individual was constructed by using the risk estimate (transformed by natural log; *b*_*n*_) from the published study and multiplying it by the number of risk alleles (*x*_*n*_: 0 (no risk allele), 1 (1 risk allele), 2 (2 risk alleles)) for each SNP as previously published (Pechlivanis et al. 2020). The products were then summed and divided by the number of SNPs (n = 100):

$$\text{weighted GRS}=\frac{{b}_{1}{x}_{1}+ {b}_{2}{x}_{2}+ \dots + {b}_{n}{x}_{n}}{n}$$

If the genotype in the score for a particular individual was missing, then the expected value was imputed based on the sample allele frequency. To calculate the GRS, the allelic scoring routine in PLINK was used (Purcell et al. 2007). The mean (0.0498) and standard deviation (SD 0.005) of the study population were used to standardize the GRS to have a mean of 0 and unit variance. Genetic risk was then analyzed per SD of the standardized GRS.

### Assessment of coronary artery calcification at baseline and first follow-up

A nonenhanced electron-beam scan with a C-100 or C-150 scanner (GE Imatron, San Francisco, CA, USA) was used to assess the CAC_b_. (Schmermund et al. 2002). The CAC_5y_ computer tomography (CT) was performed at the Radiology Department of the Alfred Krupp-Hospital, Essen with a C-150 scanner (Erbel et al. 2014; Lehmann et al. 2014). Prospective ECG triggering was performed at 80% of the RR interval, and contiguous 3-mm-thick slices from the pulmonary bifurcation to the apex of the heart were obtained in both scans at an image acquisition time of 100 ms (Lehmann et al. 2016). The methods of Agatston et al. were used to determine the CAC score (Agatston et al. 1990). The total CAC score was computed, comprising all calcified lesions in the coronary artery system. Analyses were performed using a Virtuoso workstation (Siemens Medical Solutions, Forchheim, Germany). CT scan results were not disclosed to the participants or to the study center. As previously reported, a reassessment of CAC scoring was implemented when extreme progression or regression from baseline to the 5-year examination was found (CAC_b_ ≤ 10 to CAC_5y_ > 50, CAC_b_ > 20 to CAC_5y_ ≤ 10, or otherwise, > 30% or <  − 7% annual change), accounting for the reproducibility by the given correction factors (R. C. Detrano et al. 2005; Lehmann et al. 2016). A reader with several years of experience in the evaluation of cardiac CT, who was blinded to the results of the initial reading as well as the risk factor profile of the participants, performed a second reading of the CAC score in two hundred forty-four cases. At the end, the images of both CT examinations were re-evaluated offline using the same workstation (Aquarius, TerraRecon, Foster City, CA, USA) (Lehmann et al. 2016). We used the log_e_ transformation of the CAC score plus 1, as previously suggested by Detrano et al., to address the right-skewed distribution of the CAC (R. Detrano et al. 2008).

As described previously, CAC_5y_ was predicted exponentially from the age-specific and sex-specific CAC percentiles at baseline (Erbel et al. 2014). The observed CAC_5y_ was then compared with the predicted CAC_5y_. The values within the predefined acceptance range (20% of the observed CAC_5y_ around the individually predicted CAC_5y_) were classified as “expected progression,” values above that range were classified as “rapid progression,” and values below that range as “slow progression.” For our analysis, we used a binary outcome, classifying CAC progression as either rapid or expected/slow.

### Assessment of cardiovascular risk factors

The CVD risk factors were evaluated at baseline. Body mass index (BMI) was measured as weight divided by height squared (kg/m^2^). Smoking status (current, past, and non-smokers) was evaluated as described previously (Lehmann et al. 2014). All of the participants were queried about their regular use of cardiovascular medication. The current use of medication was then recorded by means of a computer-based system with a barcode scanner. The information regarding the use of antihypertensive and HMGRI medication (ATC code: C10AA) was recorded.

The resting blood pressure was measured using an automated oscillometric blood pressure device (Omron, HEM-705CP-E) with the participants seated. The mean of the second and third values of the three measurements was calculated (Stang et al. 2006). Standardized enzymatic methods were used to determine serum triglycerides, low-density lipoprotein (LDL) cholesterol, and high-density lipoprotein (HDL) cholesterol values (ADVIA 1650, Siemens Medical Solutions, Erlangen, Germany) (Lehmann et al. 2018). DM was defined as meeting any of the following four criteria: (1) the participants reported a history of clinically diagnosed diabetes, (2) the participants took glucose-lowering drugs (ATC code: A10), (3) the participants had fasting glucose levels of greater than 125 mg/dL, or (4) the participants had nonfasting glucose levels of 200 mg/dL or greater. Socioeconomic status was defined by combining school and vocational training as total years of formal education according to the International Standard Classification of Education (UNESCO 1997) and categorized into two groups (≤ 13 vs. > 13 years).

### Statistical methods

The continuous data are presented as the mean ± SD or median (first quartile, Q1; third quartile, Q3) if the distributions of the data were substantially skewed. The count data are presented as frequencies and percentages. We first assessed the influence of DM GRS as well as each of the DM SNPs on the rapid progression of CAC adjusting for age, sex, and log(CAC_b_ + 1) (model 1: rapid progression of CAC = DM GRS/SNP + age + sex + log(CAC_b_ + 1)). Next, the influence of intake of HMGRIs on rapid progression of CAC adjusting for age, sex, and log(CAC_b_ + 1) was assessed (model 2: rapid progression of CAC = HMGRI intake + age + sex + log(CAC_b_ + 1)). Furthermore, an interaction between HMGRI intake and DM GRS/SNP on the progression of CAC (model 3: rapid progression of CAC = DM GRS/SNP + age + sex + log(CAC_b_ + 1) + HMGRI intake + HMGRI intake × DM GRS) was assessed. We further stratified the analyses by the use of HMGRI. Log-linear regression was used, adjusted for age, sex, and log(CAC_b_ + 1), to estimate the relative risks (RR) and 95% confidence interval (95% CI) (Spiegelman & Hertzmark 2005). For all the analyses, we have reported the main effect of the DM GRS/SNPs (model 1) as well as DM GRS × HMGRI intake interaction terms (model 3) on rapid progression of CAC.

Multiple testing at 5% was done for the two main questions regarding the interaction between HMGRI intake and DM GRS (HMGRI intake × DM GRS) as well as HMGRI intake × DM SNPs with progression of CAC adjusting of age, sex, and log(CAC_b_ + 1). We corrected consequently for 101 tests that translate into α_BF_ = 0.0005 using the Bonferroni procedure.

Analyses were performed using Plink v.19 (https://www.cog-genomics.org/plink2) (Purcell et al. 2007) and SAS v.9.4 (SAS Institute, Cary, NC, USA).

## Results

### Characteristics of the study population

Table 1 shows the clinical and demographic characteristics of the Heinz Nixdorf Recall Study population. During the median follow-up time of 5.1 years, the prevalence of rapid CAC progression in the HNR Study was 19.6%. Participants with HMGRI intake (N (%): 229 (7.3)), compared with non-HMGRI users (N (%): 2928 (92.7%)), were older (mean ± SD: 61.9 ± 6.9 vs. 58.7 ± 7.5 years), had more often diabetes (17.9% vs. 10.9%), and reported greater use of antihypertensive medication (55.5% vs. 29.2%). Similarly, participants with HMGRI intake had lower levels of LDL cholesterol, and higher levels of CAC at baseline and at first follow-up (Table 1). Additionally, Table 2 shows the names and defined daily doses of the HMGRI drugs used. Almost 45% of the participants used atorvastatin followed by simvastatin (26.2%), fluvastatin (12.2%), pravastatin (6.6%), lovastatin (5.7%), and cerivastatin (4.4%). The weighted DM GRSs in the HMGRI users (0.0497 ± 0.0047) and non-HMGRI users (0.0499 ± 0.005) were similar (Table 1).

**Table 1:** Basic characteristics of the study population stratified by the use of HMGRIs

	All N = 3157	Use of HMGRIs N = 229	No use of HMGRIs N = 2928
Age (years) *	59.0 ± 7.5	61.9 ± 6.9	58.7 ± 7.5
Women	1675 (53.1)	118 (51.5)	1557 (53.2)
Body mass index (kg/m^2^) *	27.6 ± 4.3	27.9 ± 3.4	27.6 ± 4.4
Diabetes	359 (11.4)	41 (17.9)	318 (10.9)
Diastolic blood pressure (mmHg) *	81.2 ± 10.6	80.4 ± 9.2	81.3 ± 10.7
Systolic blood pressure (mmHg) *	131.4 ± 19.9	133.6 ± 17.7	131.3 ± 20.1
Antihypertension medication	981 (31.1)	127 (55.5)	854 (29.2)
LDL cholesterol (mg/dL) *	146.3 ± 35.7	129.9 ± 30.2	147.6 ± 35.8
HDL cholesterol (mg/dL) *	59.3 ± 16.9	58.7 ± 17.4	59.3 ± 16.8
Triglycerides (mg/dL) †	121.0 (87.0; 174.0)	138 (100.0; 204.0)	120 (86; 171)
Total cholesterol (mg/dL) *	230.9 ± 38.3	217.2 ± 34.9	232.0 ± 38.3
Non-smoker	1377 (43.6)	103 (45.0)	1274 (43.5)
Past smoker	1080 (34.2)	80 (34.9)	1000 (34.2)
Current smoker	700 (22.2)	46 (20.1)	654 (22.3)
SES (≤ 13 years)	1086 (34.4)	82 (35.8)	1004 (34.3)
CAC score at baseline †	7.5 (0.0; 98.8)	61.4 (2.8; 279.8)	6.2 (0; 84)
CAC score at first follow-up †	27.9 (0; 202.2)	142.9 (22.4; 561.9)	24 (0; 182.3)
Slow progression of CAC	392 (12.4)	16 (7.0)	376 (12.8)
Expected progression of CAC	2147 (68.0)	143 (62.5)	2004 (68.4)
Rapid progression of CAC	618 (19.6)	70 (30.6)	548 (18.7)
Weighted GRS *	0.0498 ± 0.005	0.0497 ± 0.0047	0.0499 ± 0.005

*LDL* low-density lipoprotein, *HDL* high-density lipoprotein, *SES* socioeconomic status, *CAC* coronary artery calcification, *GRS* genetic risk score. Data are given as number (percentage) unless otherwise indicated. * Data are given as the mean ± SD. ^†^ Data are given as the median (Q1; Q3).

**Table 2:** The type and defined daily doses of HMGRI used by the study participants

ATC	Name of the durg	N (%)	Defined daily doses N (%)				
			0.5	1	1.5	2	5
C10AA01	Simvastatin	60 (26.2)	18 (30.5)	40 (67.8)	0	0	1 (1.7)
C10AA02	Lovastatin	13 (5.7)	1 (7.7)	10 (76.9)	0	1 (7.7)	1 (7.7)
C10AA03	Pravastatin	15 (6.6)	3 (20.0)	12 (80.0)	0	0	NA
C10AA04	Fluvastatin	28 (12.2)	2 (7.4)	25 (95.6)	0	0	NA
C10AA05	Atorvastatin	103 (44.9)	23 (22.3)	78 (75.7)	2 (1.9)	0	0
C10AA06	Cerivastatin	10 (4.4)	10 (100.0)	0	0	0	0

Table 3 describes all of the SNPs that are included in the GRS, indicating the chromosome number, the base pair position, nearby gene, risk allele/other allele, and risk allele frequency (RAF ≥ 3%), as well as the HWE. All the SNPs were in HWE (*p* > 0.01) except rs495828 (*p* = 0.003).

**Table 3:** Relative risk for the rapid progression of CAC with either the DM GRS/the individual SNPs alone as risk factors or the interaction between HMGRI intake and the DM GRS/SNPs as risk factors

Genetic risk score/SNP	Chr	Base position	Gene	Risk/other allele	RAF	HWE	Model 1: Rapid progression of CAC = DM GRS/SNP + age + sex + log(CAC_b_ + 1)	Model 3: Rapid progression of CAC = DM GRS/SNP + age + sex + log(CAC_b_ + 1) + HMGRI intake + DM GRS/SNP × HMGRI
							RR [95% CI], *p*	RR [95% CI], *p*
Diabetes mellitus genetic risk score							1.01 [0.94; 1.10], 0.73	1.08 [0.83; 1.41], 0.56
rs17106184	1	50,909,985	*FAF1*	G/A	0.91	1	1.13 [0.92; 1.39], 0.25	0.88 [0.45; 1.75], 0.72
rs10923931	1	120,517,959	*NOTCH2*	T/G	0.10	0.63	0.97 [0.80; 1.17], 0.71	1.06 [0.57; 1.96], 0.86
rs2075423	1	214,154,719	*PROX1*	G/T	0.63	0.82	0.97 [0.87; 1.09], 0.67	1.18 [0.81; 1.72], 0.40
rs2867125	2	622,827	*TMEM18*	C/T	0.82	0.95	1.10 [0.94; 1.28], 0.25	0.78 [0.49; 1.26], 0.31
rs780094	2	27,741,237	*GCKR*	C/T	0.60	0.26	0.99 [0.88; 1.11], 0.87	1.27 [0.88; 1.83], 0.20
rs10203174	2	43,690,030	*THADA*	C/T	0.88	0.73	0.84 [0.72; 0.99], 0.04	1.23 [0.67; 2.26], 0.51
rs243088	2	60,568,745	*BCL11A*	T/A	0.46	0.20	0.99 [0.89; 1.11], 0.90	1.17 [0.83; 1.66], 0.36
rs11123406	2	111,950,541	*BCL2L11*	T/C	0.35	0.17	1.04 [0.92; 1.18], 0.55	0.99 [0.68; 1.45], 0.96
rs998451	2	135,429,288	*TMEM163*	A/G	0.41	0.14	1.00 [0.90; 1.12], 0.94	1.15 [0.80; 1.65], 0.45
rs4410242	2	161,192,070	*RBMS1*	G/A	0.81	0.69	0.99 [0.86; 1.14], 0.84	1.10 [0.70; 1.73], 0.68
rs3923113	2	165,501,849	*GRB14*	A/C	0.61	0.76	0.92 [0.82; 1.04], 0.18	0.96 [0.66; 1.40], 0.85
rs2943640	2	227,093,585	*IRS1*	C/A	0.64	0.21	1.00 [0.89; 1.12], 0.97	1.15 [0.79; 1.67], 0.46
rs1801282	3	12,393,125	*PPARG*	C/G	0.86	0.27	1.01 [0.87; 1.19], 0.85	0.86 [0.54; 1.36], 0.51
rs7612463	3	23,336,450	*UBE2E2*	C/A	0.90	0.84	1.09 [0.90; 1.33], 0.38	0.66 [0.37; 1.16], 0.15
rs831571	3	64,048,297	*PSMD6*	C/T	0.82	0.76	0.96 [0.83; 1.12], 0.63	1.32 [0.80; 2.18], 0.28
rs6795735	3	64,705,365	*ADAMTS9*	C/T	0.59	0.79	1.02 [0.91; 1.14], 0.78	1.30 [0.89; 1.91], 0.18
rs11717195	3	123,082,398	*ADCY5*	T/C	0.78	0.84	0.94 [0.83; 1.08], 0.41	1.04 [0.68; 1.59], 0.85
rs4402960	3	185,511,687	*IGF2BP2*	T/G	0.31	0.29	1.02 [0.90; 1.15], 0.76	1.15 [0.78; 1.71], 0.48
rs16861329	3	186,666,461	*ST64GAL1*	C/T	0.87	0.42	1.08 [0.90; 1.28], 0.42	0.96 [0.56; 1.65], 0.89
rs6808574	3	187,740,523	*LPP*	C/T	0.61	0.48	1.07 [0.99; 1.21], 0.27	0.74 [0.51; 1.09], 0.12
rs4458523	4	6,289,986	*WFS1*	G/T	0.60	0.88	1.06 [0.94; 1.18], 0.36	1.11 [0.77; 1.58], 0.58
rs7674212	4	103,988,899	*CISD2*	G/T	0.58	0.79	0.89 [0.79; 1.00], 0.04	1.22 [0.85; 1.78], 0.28
rs2706785	4	122,660,250	*TMEM155*	G/A	0.03	0.12	0.75 [0.51; 1.10], 0.14	1.37 [0.40; 4.69], 0.62
rs6813195	4	153,520,475	*TMEM154*	C/T	0.73	1	1.00 [0.88; 1.14], 0.96	0.89 [0.58; 1.36], 0.60
rs1996546	4	185,714,289	*ACSL1*	G/T	0.85	0.24	0.98 [0.83; 1.14], 0.76	1.52 [0.88; 2.61], 0.13
rs702634	5	53,271,420	*ARL15*	A/G	0.68	0.16	1.00 [0.89; 1.13], 0.96	0.97 [0.66; 1.43], 0.87
rs459193	5	55,806,751	*ANKRD55*	G/A	0.74	0.55	1.07 [0.94; 1.21], 0.32	0.95 [0.61; 1.48], 0.82
rs6878122	5	76,427,311	*ZBED3*	G/A	0.29	0.72	0.98 [0.86; 1.11], 0.74	0.66 [0.42; 1.02], 0.06
rs329122	5	133,864,599	*PHF15*	A/G	0.40	0.88	1.00 [0.89; 1.12], 0.95	0.83 [0.56; 1.23], 0.35
rs9505118	6	7,290,437	*SSR1/RREB1*	A/G	0.60	0.19	1.02 [0.91; 1.14], 0.71	1.06 [0.74; 1.51], 0.75
rs7756992	6	20,679,709	*CDKAL1*	G/A	0.29	0.40	1.00 [0.88; 1.13], 0.98	0.82 [0.53; 1.29], 0.40
rs3130501	6	31,136,453	*POU5F1/TCF19*	G/A	0.73	0.03	0.95 [0.84; 1.08], 0.45	0.97 [0.66; 1.44], 0.89
rs2050188	6	32,339,897	*HLA-DRB5*	T/C	0.63	0.81	0.99 [0.88; 1.12], 0.88	1.02 [0.69; 1.50], 0.92
rs9271775	6	32,594,328	*HLA-DQA1*	T/C	0.82	0.14	1.04 [0.90; 1.21], 0.57	1.49 [0.85; 2.59], 0.16
rs9470794	6	38,106,844	*ZFAND3*	T/C	0.92	0.53	1.16 [0.92; 1.46], 0.21	0.50 [0.28; 0.92], 0.02
rs4407733	6	137,299,152	*IL20RA*	A/G	0.53	0.91	0.94 [0.84; 1.05], 0.24	1.30 [0.91; 1.85], 0.15
rs622217	6	160,766,770	*SLC22A3*	T/C	0.47	0.50	1.04 [0.93; 1.16], 0.54	1.06 [0.75; 1.49], 0.76
rs17168486	7	14,898,282	*DGKB*	T/C	0.18	0.95	0.90 [0.77; 1.04], 0.16	1.03 [0.62; 1.69], 0.91
rs849135	7	28,196,413	*JAZF1*	G/A	0.50	0.86	1.06 [0.94; 1.18], 0.34	0.98 [0.69; 1.38], 0.89
rs10278336	7	44,245,363	*GCK*	A/G	0.58	0.26	1.11 [0.99; 1.24], 0.08	1.09 [0.75; 1.60], 0.64
rs6467136	7	127,164,958	*GCC1*	A/G	0.46	0.46	1.01 [0.90; 1.13], 0.87	1.13 [0.77; 1.64], 0.54
rs13233731	7	130,437,689	*KLF14*	G/A	0.51	0.22	1.04 [0.93; 1.16], 0.53	1.09 [0.77; 1.55], 0.63
rs9648716	7	140,612,163	*BRAF*	T/A	0.14	0.94	1.02 [0.87; 1.20], 0.80	0.97 [0.59; 1.60], 0.92
rs1182397	7	157,031,407	*MNX1*	G/T	0.84	0.69	1.00 [0.85; 1.16], 0.97	0.81 [0.48; 1.34], 0.41
rs12681990	8	36,859,186	*KCNU1*	C/T	0.19	0.55	0.97 [0.84; 1.13], 0.71	0.82 [0.49; 1.39], 0.47
rs516946	8	41,519,248	*ANK1*	C/T	0.76	0.03	1.04 [0.91; 1.18], 0.55	1.44 [0.91; 2.28], 0.12
rs7845219	8	95,937,502	*TP53INP1*	T/C	0.54	0.94	1.02 [0.91; 1.14], 0.75	0.96 [0.67; 1.37], 0.83
rs3802177	8	118,185,025	*SLC30A8*	G/A	0.69	0.55	1.04 [0.92; 1.18], 0.49	1.21 [0.81; 1.81], 0.36
rs7041847	9	4,287,466	*GLIS3*	A/G	0.51	0.45	0.91 [0.80; 1.01], 0.08	0.81 [0.56; 1.17], 0.27
rs17584499	9	8,879,118	*PTPRD*	T/C	0.20	0.43	1.09 [0.95; 1.26], 0.21	1.53 [0.96; 2.44], 0.07
rs10811661	9	22,134,094	*CDKN2A/B*	T/C	0.83	0.61	1.08 [0.92; 1.25], 0.34	1.02 [0.61; 1.70], 0.94
rs17791513	9	81,905,590	*TLE4*	A/G	0.93	0.56	1.07 [0.85; 1.36], 0.56	0.90 [0.44; 1.84], 0.76
rs2796441	9	84,308,948	*TLE1*	G/A	0.60	0.47	1.01 [0.90; 1.13], 0.92	0.96 [0.67; 1.40], 0.85
rs495828	9	136,154,867	*ABO*	T/G	0.24	0.003	0.92 [0.80; 1.05], 0.21	0.89 [0.57; 1.38], 0.59
rs11257655	10	12,307,894	*CDC123*	T/C	0.21	0.41	0.95 [0.83; 1.10], 0.52	0.80 [0.50; 1.28], 0.36
rs1802295	10	70,931,474	*VPS26A*	T/C	0.32	0.02	1.01 [0.90; 1.13], 0.88	0.97 [0.67; 1.42], 0.89
rs12571751	10	80,942,631	*ZMIZ1*	A/G	0.53	0.11	0.99 [0.88; 1.10], 0.79	0.95 [0.66; 1.38], 0.80
rs1111875	10	94,462,882	*HHEX/IDE*	C/T	0.59	0.88	0.98 [0.88; 1.10], 0.77	1.14 [0.79; 1.66], 0.49
rs7903146	10	114,758,349	*TCF7L2*	T/C	0.28	0.89	0.93 [0.82; 1.06], 0.27	0.77 [0.51; 1.18], 0.22
rs10886471	10	121,149,403	*GRK5*	T/C	0.45	0.48	1.02 [0.90; 1.14], 0.78	0.68 [0.46; 1.01], 0.05
rs2421016	10	124,167,512	*PLEKHA1*	C/T	0.50	0.61	0.89 [0.80; 1.00], 0.06	1.03 [0.72; 1.48], 0.87
rs2334499	11	1,696,849	*DUSP8*	T/C	0.40	0.82	1.04 [0.93; 1.16], 0.48	1.09 [0.77; 1.55], 0.61
rs163184	11	2,847,069	*KCNQ1*	G/T	0.50	0.72	0.98 [0.88; 1.10], 0.74	1.11 [0.78; 1.60], 0.55
rs5215	11	17,408,630	*KCNJ11*	C/T	0.36	0.51	1.03 [0.92; 1.16], 0.62	1.01 [0.70; 1.45], 0.95
rs3736505	11	43,876,435	*HSD17B12*	G/A	0.30	0.25	1.00 [0.88; 1.13], 0.98	1.00 [0.68; 1.48], 0.99
rs11227234	11	65,365,171	*MAP3K11*	T/G	0.24	0.11	1.01 [0.88; 1.15], 0.88	1.27 [0.84; 1.92], 0.26
rs1552224	11	72,433,098	*ARAP1 (CENTD2)*	A/C	0.84	0.79	0.78 [0.68; 0.90], 0.001	0.94 [0.60; 1.47], 0.80
rs10830963	11	92,708,710	*MTNR1B*	G/C	0.29	0.36	1.09 [0.96; 1.23], 0.19	1.04 [0.72; 1.51], 0.84
rs11063069	12	4,374,373	*CCND2*	G/A	0.21	0.79	1.04 [0.90; 1.19], 0.61	1.00 [0.65; 1.56], 0.99
rs10842994	12	27,965,150	*KLHDC5*	C/T	0.79	0.48	0.97 [0.85; 1.11], 0.67	0.56 [0.34; 0.91], 0.02
rs2261181	12	66,212,318	*HMGA2*	T/C	0.09	0.51	1.00 [0.82; 1.22], 0.99	0.69 [0.35; 1.39], 0.30
rs7955901	12	71,433,293	*TSPAN8*	C/T	0.45	0.36	1.06 [0.95; 1.19], 0.31	0.75 [0.52; 1.09], 0.13
rs12427353	12	121,426,901	*HNF1B*	G/C	0.82	0.81	0.99 [0.86; 1.18], 0.91	0.82 [0.52; 1.29], 0.38
rs1727294	12	123,616,514	*MPHOSPH9*	G/A	0.79	0.87	0.92 [0.80; 1.05], 0.20	1.16 [0.75; 1.79], 0.50
rs825476	12	124,568,456	*CCDC92*	T/C	0.57	0.71	1.06 [0.94; 1.19], 0.31	1.00 [0.69; 1.43], 0.99
rs10507349	13	26,781,528	*RNF6*	G/A	0.77	0.32	0.92 [0.81; 1.06], 0.25	1.00 [0.65; 1.55], 0.99
rs576674	13	33,554,302	*KL*	G/A	0.17	0.79	0.98 [0.84; 1.14], 0.78	0.79 [0.46; 1.32], 0.35
rs1359790	13	80,717,156	*SPRY2*	G/A	0.72	1	1.02 [0.90; 1.16], 0.73	0.98 [0.66; 1.47], 0.93
rs7985179	13	91,940,169	*MIR17HG*	T/A	0.75	0.52	0.91 [0.80; 1.04], 0.17	1.06 [0.69; 1.63], 0.78
rs17109256	14	79,939,993	*NRXN3*	A/G	0.22	0.50	0.97 [0.85; 1.11], 0.67	1.41 [0.93; 2.13], 0.10
rs7403531	15	38,822,905	*RASGRP1*	T/C	0.21	0.44	1.05 [0.90; 1.20], 0.54	1.07 [0.70; 1.63], 0.77
rs4502156	15	62,383,155	*C2CD4A*	C/T	0.41	0.88	0.92 [0.82; 1.03], 0.14	1.18 [0.81; 1.73], 0.39
rs7178572	15	77,747,190	*HMG20A*	G/A	0.70	1	1.04 [0.92; 1.18], 0.53	0.91 [0.61; 1.36], 0.66
rs11634397	15	80,432,222	*ZFAND6*	G/A	0.68	0.65	0.96 [0.85; 1.08], 0.53	0.93 [0.64; 1.37], 0.72
rs2028299	15	90,374,257	*AP3S2*	C/A	0.28	0.78	1.02 [0.89; 1.15], 0.82	1.02 [0.67; 1.54], 0.94
rs12899811	15	91,544,076	*PRC1*	G/A	0.31	0.97	1.04 [0.92; 1.17], 0.54	0.81 [0.54; 1.20], 0.29
rs9940149	16	300,641	*ITFG3*	G/A	0.81	0.11	0.87 [0.75; 1.00], 0.05	1.34 [0.83; 2.16], 0.24
rs9936385	16	53,819,169	*FTO*	C/T	0.41	0.88	0.94 [0.84; 1.06], 0.31	1.09 [0.77; 1.55], 0.61
rs7202877	16	75,247,245	*BCAR1*	T/G	0.89	0.71	0.92 [0.77; 1.10], 0.37	0.97 [0.54; 1.73], 0.91
rs2925979	16	81,534,790	*CMIP*	T/C	0.30	0.67	1.03 [0.91; 1.17], 0.60	1.22 [0.84; 1.79], 0.30
rs391300	17	2,216,258	*SRR*	C/T	0.64	0.67	1.02 [0.91; 1.14], 0.77	0.87 [0.59; 1.29], 0.48
rs8068804	17	3,985,864	*ZZEF1*	A/G	0.32	0.07	1.01 [0.89; 1.13], 0.93	1.45 [1.01; 2.09], 0.04
rs17676067	17	9,791,375	*GLP2R*	C/T	0.28	0.12	0.98 [0.86; 1.10], 0.70	1.27 [0.89; 1.81], 0.18
rs11651052	17	36,102,381	*HNF1B*	G/A	0.53	0.33	1.07 [0.96; 1.20], 0.21	0.94 [0.66; 1.34], 0.74
rs15563	17	47,005,193	*GIP*	G/A	0.53	0.61	1.04 [0.93; 1.16], 0.53	1.00 [0.71; 1.40], 0.98
rs12970134	18	57,884,750	*MC4R*	A/G	0.26	0.82	0.94 [0.83; 1.07], 0.39	1.37 [0.92; 2.06], 0.12
rs10401969	19	19,407,718	*CILP2*	C/T	0.08	0.52	1.22 [1.00; 1.48], 0.04	1.21 [0.63; 2.34], 0.57
rs3786897	19	33,893,008	*PEPD*	A/G	0.58	0.24	0.95 [0.85; 1.06], 0.33	0.99 [0.69; 1.43], 0.96
rs8108269	19	46,158,513	*GIPR*	G/T	0.31	0.70	0.93 [0.83; 1.06], 0.28	0.78 [0.51; 1.19], 0.25
rs4812829	20	42,989,267	*HNF4A*	A/G	0.18	0.67	1.02 [0.88; 1.19], 0.75	1.04 [0.66; 1.62], 0.87

*Chr* chromosome, *Gene* specify the nearby gene, *RAF* risk allele frequency, *HWE* Hardy–Weinberg equilibrium, *CAC*_*b*_ coronary artery calcification score at baseline. The model is adjusted for age, sex, and log(CAC_b_ + 1) and consists of the interaction between GRS/SNP × HMGRI intake.

Looking at the main effect, in the age, sex, and log(CAC_b_ + 1)-adjusted model, no association of the weighted DM GRS with the rapid progression of CAC was observed (model 1: RR per SD increase in the DM GRS [95% CI]: 1.01 [0.94; 1.10]) (Table 3). Table 3 further shows the association of individual SNPs with the rapid progression of CAC. Although a genetic association at a nominal significance level with rapid CAC progression was observed for five SNPs (rs10203174 (*THADA*), rs7674212 (*CISD2*), rs1552224 (*ARAP1 (CENTD2)*), rs9940149 (*ITFG3*), and rs10401969 (*CILP2*)) (Table 3), after correction for multiple testing, none of the SNPs was significantly associated.

As almost twofold odds of HMGRI intake for CAC progression are already published for the HNR study population, we re-analyzed the current dataset (Dykun et al. 2016). The main effect of HMGRI intake on rapid progression of CAC in the model adjusting for age, sex, and log(CAC_b_ + 1) showed similar significant effect (model 2: 1.64 [1.27; 2.11], *p* = 0.0001) (data not shown).

Furthermore, looking at the interaction of DM GRS × HMGRI intake on rapid progression of CAC, no indication of interaction was found after multiple testing (model 3: 1.08 [0.83; 1.41]) (Table 3). Table 3 further shows the interaction of individual SNP × HMGRI intake on rapid progression of CAC. Indication of SNP × HMGRI intake interaction was not observed for any of the five SNPs that appeared to be associated with CAC progression at nominal significance level (rs10203174, rs7674212, rs1552224, rs9940149, and rs10401969) (Table 3). Apart from that, SNP × HMGRI intake interactions at the nominal significance level were observed for four other SNPs (rs9470794 (*ZFAND3*), rs10886471 (*GRK5*), rs10842994 (*KLHDC5*), and rs8068804 (*ZZEF1*)) (Table 3). However, none of the SNP × HMGRI intake interactions remained significant after correcting for multiple testing (*p* < 0.0005).

Looking in the HMGRI intake stratified groups, although the group taking HMGRIs showed higher effect size for the DM GRS on the rapid progression of CAC (1.09 [0.85; 1.40]), this effect was not statistically significant. The group without HMGRI intake showed no significant influence of DM GRS on the rapid progression of CAC either (1.01 [0.93; 1.10]) (Table 4).

**Table 4:** Association of the diabetes mellitus–associated genetic risk score in the stratum with HMGRI intake at baseline

Genetic risk score	Rapid progression of CAC RR [95%CI], *p*
HMGRI intake = Yes	
Diabetes mellitus genetic risk score	1.09 [0.85; 1.40], 0.48
HMGRI intake = No	
Diabetes mellitus genetic risk score	1.01 [0.93; 1.10], 0.85

*CAC*_*b*_ coronary artery calcification score at baseline. The model is adjusted for age, sex, and log(CAC_b_ + 1).

## Discussion and conclusion

Analyzing data from the population-based Heinz Nixdorf Recall study, we investigated for the first time the influence that results from the interplay of both a diabetes mellitus genetic risk score (DM GRS; constructed using GWAS-identified diabetes-associated genetic variants) and individual SNPs with HMGRI intake on the progression of CAC (Table 5). We did not find any evidence of an association of CAC progression with DM GRS alone nor with the interaction of DM GRS and HMGRI intake. The interaction of individual SNPs and HMGRI intake was not correlated with CAC progression either.

**Table 5:** Comparison of the present study and related studies

	Participants	Endpoint	Assessed factors	Average follow-up time
Houslay, 2006	54 placebo 48 atorvastatin, 80 mg	CAC progression Serum CRP concentration Serum LDL cholesterol concentration	• HMGRI intake	2 years
Anand, 2007	402 (observational)	CAC progression	• Demographic data • Risk factors • Glycemic control • Medication (including HMGRI intake) • Serum hs-CRP • IL-6 • Plasma OPG	2,5 years
Terry, 2007	40 placebo 40 simvastatin, 80 mg	CAC progression AAC progression	• HMGRI intake	1 year
Saremi, 2012	197 T2DM patients (observational)	CAC progression AAC progression	• HMGRI intake	4,6 years
Puri, 2015	3495 (observational)	CaI Coronary PAV	• HMGRI intake	1,5 – 2 years
Rhee, 2015	19,920 (observational)	CAC score	Eligibility for HMGRI according to: • ACC/AHA guideline • ATPIII guideline	single measurement/non follow-up
Warters, 2011	3797 atorvastatin, 10 mg 3798 atorvastatin, 80 mg 3724 simvastatin, 20 mg 3737 atorvastatin, 80 mg 1898 placebo 1905 atorvastatin, 80 mg	New-onset T2DM Major cardiovascular events	• Fasting glucose levels	4,9 years
			• Triglyceride levels	
			• BMI	4,8 years
			• History of hypertension	
			• HMGRI intake	4,9 years
Carter, 2013	38,470 pravastatin 268,254 atorvastatin 5636 fluvastatin 6287 lovastatin 76,774 rosuvastatin 75,829 simvastatin	New-onset T2DM	• HMGRI intake	5 years
Cederberg, 2015	8749 (observational)	New-onset T2DM	• HMGRI intake	5,9 years
Snell-Bergeon, 2003	109 T1DM patients (observational)	CAC progression	• Demographic data • Glycemic control • Baseline CAC • BMI and insulin interaction	2,7 years
Koulaouzidis, 2013	388 with CAC score of 0 at baseline	CAC progression	• Demographic data • BMI • DM • Smoking • Hypertension • Hypercholesterolemia	1 – 6 years
Henein, 2015	419 placebo 432 atorvastatin, 20 mg 164 atorvastatin, 10 mg 179 atorvastatin, 80 mg	CAC progression	• HMGRI intake	2 years, 4 years, and 6 years 1 year
Dykun, 2016	3483 (observational)	CAC progression	• HMGRI intake	5 years
Present study (Pechlivanis, 2021)	3157 (observational)	CAC progression	• **DM-associated SNPs** • **DM GRS** • **Interaction of DM SNPs × HMGRI intake** • **Interaction of DM GRS × HMGRI intake**	5 years

*AAC* abdominal aortic artery calcification, *ACC/AHA* American College of Cardiology/American Heart Association, *ATPIII* Adult Treatment Panel III, *BMI* body mass index, *CAC* coronary artery calcification, *CaI* calcium indices, *CRP* serum C-reactive protein, *(T1/T2) DM* (type 1/type 2) diabetes mellitus, *GRS* genetic risk score, *LDL* low-density lipoprotein, *PAV* percent atheroma volume, *SNP* single nucleotide polymorphism.

HMGRIs are typically used to reduce LDL cholesterol levels in order to prevent CHD. They are generally considered safe and have even been shown to enhance life expectancy (Jacobs et al. 2013), but due to their pleiotropic effects, their use can have unexpected consequences, such as increased progression of CAC or increased risk of developing DM. There is evidence from several studies that the use of HMGRIs (especially in diabetic patients) leads to a greater degree of progression of CAC (Anand et al., 2007; Houslay et al. 2006; Saremi et al. 2012; Terry et al. 2007). In accordance with those results, Dykun et al*.* already showed that the prevalence of rapid progression of CAC and cardiovascular risk factors in the HNR study was higher among HMGRI users than among non-HMGRI users (Dykun et al. 2016).

As our analyses used the data from the same cohort, we could also see that HMGRIs promote CAC progression irrespective of the DM GRS. However according to our research question, our study did not reveal any impact of the individual diabetes-associated genetic variants that had previously been identified via diabetes-associated GWAS on the progression of CAC. Neither was CAC progression affected by a DM GRS that included the entirety of these 100 SNPs. Furthermore, the interaction of DM GRS × HMGRI intake was also not significantly associated with CAC progression. These results suggest that the association of DM and CAC progression is mediated by factors other than a genetic disposition to diabetes. Further investigations will be needed to determine whether this association is due to poor glycemic control, as suggested by Snell-Bergeon et al., or other causes like DM-associated lifestyle factors.

Importantly, these negative results persisted upon additionally taking into account the participants’ HMGRI intake status, which shows that DM GRS does not statistically significantly affect CAC progression. This can be judged as a point in favor of the common practice of treating patients with increased CHD risk with HMGRIs—even those with an increased genetic disposition for developing DM and despite a growing body of evidence that HMGRIs can also promote the progression of CAC. Presently, we witness both widespread use of HMGRIs and a globally increasing prevalence of DM and CHD. Considering this, an acceleration of CAC progression due to the interaction of HMGRIs and DM-associated genetic variants could have represented a substantial hidden risk factor for developing CHD—and presumably a considerable burden for public health. In that case, reconsideration of HMGRI medication for DM patients would likely have become necessary. However, the results of our study do not show a statistical significant influence of such an interaction on CAC progression, therefore delivering an important confirmation of current guidelines regarding HMGRI prescription.

The strength of the present study is the long follow-up time of 5 years for the assessment of CAC progression. However, we must emphasize that due to the small cohort size, especially regarding the subgroup of HMGRI users, the statistical power of the present study is limited. The lack of an association of the DM GRS could be attributed to the sample size being relatively small relative to small effect size observed for any of the individual diabetes mellitus–related SNPs which were integrated together into a genetic risk score. The small sample size has further limited the statistical power for the analyses of the individual SNPs. It is also possible that several lifestyle factors such as physical activity, consumption of alcohol, or dietary factors can modify the effect of the diabetes mellitus–related genetic variants on the rapid progression of CAC, which could be investigated in larger studies. Nevertheless, our study provides first insights into the hitherto disregarded pharmacogenetic aspects of HMGRI medication in the context of diabetes and CAC progression.

In conclusion, our study showed no accelerated progression of CAC that could be attributed to the combination of HMGRI intake and genetic DM risk factors; and thus, it corroborates the current recommendations of the EACPR and ACC/AHA regarding the use of HMGRIs. While limited in scale, it supports the prescription of HMGRIs as the preferred preemptive CHD medication in the face of new insights into the various pleiotropic effects of HMGRIs, even in individuals exhibiting an increased genetic disposition for the development of diabetes mellitus.

## Data Availability

Due to data security reasons, i.e., the data contain potentially participant identifying information, the Heinz Nixdorf Recall Study does not allow sharing data as a public use file. However, for the purpose of replication, other authors/researchers are allowed to access data upon request, which is the same way the authors of the present paper obtained the data. Data requests can be addressed to recall@uk-essen.de.
